# Indications and clinical outcomes of percutaneous cholecystostomies in acute cholecystitis: a study from Qatar

**DOI:** 10.1186/s12893-025-02765-4

**Published:** 2025-03-15

**Authors:** Mohamed Said Ghali, Syed Muhammad Ali, Khadija Jaffar Siddig Gibreal, Rajvir Singh, Mona S. Shehata, Raed M. Al-Zoubi, Ahmad Zarour

**Affiliations:** 1 Department of Surgery, Acute Care Surgery, Hamad Medical Corporation, Doha, 3050 Qatar; 2 Department of General Surgery, Ain Shams University, Cairo, Egypt; 3 Wiell Cornell medical college, Doha, Qatar; 4 Department of Pharmacy, Woman’s Wellness and Research Center, Hamad Medical Corporation, Doha, Qatar; 5 Surgical Research Section, Department of Surgery, Hamad Medical Corporation, Doha, Qatar; 6 Department of Biomedical Sciences, QU-Health, College of Health Sciences, Qatar University, Doha, 2713 Qatar; 7 Department of Chemistry, Jordan University of Science and Technology, Irbid, 22110 Jordan; 8 Department of Surgery, College of Medicine, Qatar University, Doha, 2713 Qatar

**Keywords:** Acute cholecystitis, Percutaneous cholecystostomy, Laparoscopy- cholecystectomy, Emergency

## Abstract

**Introduction:**

: Acute cholecystitis (AC) is a prevalent condition in emergency departments (EDs). Standard care involves early laparoscopic cholecystectomy; however, in cases of delayed presentation, high surgical risk, or during situations like the COVID-19 pandemic, percutaneous cholecystostomy (PC) serves as an alternative management strategy. This study reports our center’s experience with PC in managing AC, providing insights from a unique geographical context.

**Methods:**

We conducted a retrospective review of 97 patients undergoing PC operation from June 1, 2016, to January 1, 2021. The data collected included demographic details, indications for PC, clinical outcomes, ICU admissions, overall mortality, and long-term follow-up.

**Results:**

The cohort comprised 61.9% male patients with a mean age of 67.2 ± 15.5 years. The primary comorbidity was hypertension (83.5%), and 88.6% had an ASA (American Society of Anesthesiologists) score of ≥ III. The main cause of AC was calculous type, and 15.2% of cases were acalculous cholecystitis. Main Tokyo Guidelines 18 (TG 18) grade was grade II and was found in 56.4% of patients. The readmission rate was 33.1% and overall mortality rate was 34% during follow-up. The native population in Qatar were older and burdened with more co-morbidities. High risk of surgery was the main indication for PC, followed by delayed presentation of AC. Patients with delayed presentations were younger (*p* = 0.051), had higher albumin levels (*p* = 0.005), and had lower ICU admission rates (*p* = 0.002) and mortality (*p* = 0.014) than those with multiple comorbidities. The overall Mortality rates post-PC were 34%, predominantly attributed to underlying conditions rather than the PC procedure itself. Patients who proceeded to post-PC cholecystectomy were younger, had higher albumin levels, and experienced fewer readmissions (*p* < 0.05).

**Conclusion:**

In high-risk patients or when surgical risk is prohibitive, PC is a viable and effective alternative for AC management. Post-PC cholecystectomy was associated with favorable outcomes, suggesting PC as a bridge to surgery in selected patients. This study highlights the role of PC in a high-risk population within our regional setting.

## Introduction

Acute cholecystitis (AC) is one of the most common illnesses seen in the emergency department (ED). Every year, over 300,000 persons with AC are admitted to ED in the United States as the number of patients with AC increases with age. Unless there is a contraindication to perform surgery safely, the World Society of Emergency Surgery supports early surgery, which can be performed up to seven days following onset of pain [1]. 

AC is characterized by pain in the right upper quadrant, fever, and neutrophilic leukocytosis. The most common cause of inflammation is gall bladder (GB) stones due to its impaction at GB passage either in the Hartman’s area or in the cystic duct lumen; however, 5–10% of patients present with acalculous cholecystitis especially in critically ill patients on regular parenteral nutrition and more with older population. In most patients, the diagnosis of AC is established mostly on clinical evaluation, with a positive Murphy’s sign on examination confirmed by laboratory and radiological results [2]. Tokyo guidelines 2018 (TG 18) for AC were widely applied for management of cholecystitis with higher specificity and higher diagnostic accuracy [3, 4]. Whereas gallstones are one cause of chronic cholecystitis, they can cause AC in 6 to 11% of people with chronic calculous cholecystitis [5].

It is advised to perform an early laparoscopic cholecystectomy on patients with AC within one week of their complaint and presentation [6]. If the patient’s presentation was delayed for more than seven days, or if the patient has several comorbidities with a high risk of surgery, an alternate option is conservative therapy with antibiotics. If the patient’s condition improves, he will be scheduled for interval cholecystectomy, if applicable [7].

A small percentage of people who come with sepsis or antibiotic treatment fail to improve their AC sepsis, would require a less invasive procedure such as percutaneous cholecystostomy (PC), which has 91% success rate to ameliorate the symptoms [8, 9]. PC was first reported in 1980 by Radder et al. and was indicated as interval management of delayed AC but, with time, indication extended to cover elder and high surgical risk population [10].

One more indication for PC was During the coronavirus epidemic (COVID-19). As, conservative care and less invasive procedures prevailed to reduce virus transmission. Many international surgical societies advocated for avoiding surgery, particularly laparoscopy, and instead seeking alternate treatment, hence antibiotic medication with or without cholecystostomy tube insertion has important role in emergency AC management [11, 12].

This study aimed to assess the indications and clinical outcomes of PC as a management strategy for AC in a high-risk population within a unique geographical setting.

### Patients and methods

Hamad Medical Corporation is the main public health service in Qatar. it has three main General Hospital; Hamad General Hospital, Alkhor hospital and Alwakrah hospital. Study population includes all medical records of patients admitted with diagnosis of AC or inpatients admitted previously for other conditions and later was managed with PC for AC between June 1st, 2016 to January 1st, 2021 with at least one year follow up. We applied the TG 18 guidelines [13] in managing AC. For patients with TG18 severity grade I or II, we offered surgery within one week of symptom onset for those presenting as emergencies. However, for grade III or other grades with symptoms lasting more than seven days, we managed the condition non-operatively, either medically alone or with PC if medical treatment did not result in improvement. We included patients whose primary indication for PC was either delayed presentation (more than one week after symptom onset) or high-risk status due to multiple comorbidities, particularly after failure of conservative treatment. Acute cholecystitis was defined and diagnosed according to TG18, as shown in Table 1. Regarding the indication for PC, delayed cholecystectomy was defined as surgery for patients presenting more than seven days after the onset of AC symptoms, based on our department’s policy. Patients were further categorized based on age and comorbidity scores (ASA and Charlson Comorbidity Index (**CCI**), allowing for a targeted analysis of mortality and morbidity risks. High-risk patients were classified as those with an American Society of Anesthesiologists (ASA) physical status score ≥ 3 or a Charlson Comorbidity Index score ≥ 5 [14, 15]. Patients under the age of 14 (14 years is the cut off age for adult patients where our department manage only adult population) and those who had PC for a reason other than AC like cholangitis were excluded. We excluded cases performed during the COVID-19 pandemic where the reason for PC was non-surgical management of early-onset acute cholecystitis, in order to minimize the risk of COVID-19 transmission. All procedures were carried out in Hamad General Hospital as it is the main facility that has radiology set up for this procedure. Medical Research Center approved this retrospective study (**MRC-01-23-616**). This Data can be retrieved from HMC Business Intelligence Unit or radiology department. the data collected were patient’s demographic, clinical (age, gender, nationality and body mass index (BMI)), and laboratory (white blood cells (WBCs), Neutrophil count, Lymphocyte count, Platelets, hemoglobin level (HGB), international normalization ration (INR), alanine transaminase (ALT), aspartate aminotransferase (AST), alkaline phosphatase (ALP), Total Bilirubin (BIL T), serum creatinine (Cr), blood urea nitrogen (BUN), potential of hydrogen (pH), Base excess, serum potassium (K), serum sodium (NA), C reactive protein (CRP), serum lactate, serum albumin (ALB), and serum glucose) and radiological finding, comorbidities (diabetes mellitus (DM), hypertension (HTN), coronary artery disease (CAD), chronic kidney disease (CKD), Chronic Liver disease (CLD) and other comorbidities), intensive care unit (ICU) admission and stay, date of PC placement and removal, PC complications, PC related and overall mortality, length of hospital stay (LOS), readmission, follow up and redo procedure were collected from patient files. Through patient identifier health number, we retrieve all data required for this study from electronic medical record (Cerner).

**Table 1:** TG 18 diagnostic criteria for AC diagnosis

**A**. Local signs of inflammation etc.: (1) Murphy’s sign, (2) RUQ mass/pain/tenderness
**B**. Systemic signs of inflammation etc.: (1) Fever, (2) elevated CRP, (3) elevated WBC count
**C**. Imaging findings: Imaging findings characteristic of acute cholecystitis
**Suspected diagnosis**: One item in A + one item in B **Definite diagnosis**: One item in A + one item in B + C

Cited from Yokoe et al. [3].

Upon admission, patients with acute cholecystitis (AC) received supportive treatment aimed at noninvasive medical management. Patients kept fasting without oral intake with appropriate IV hydration, electrolyte adjustments, and venous thromboembolism risk assessments. The TG18 severity grading system was applied to guide our management (Table 2).

**Table 2:** TG 18 severity garding for AC

Severity	Criteria
Grade 1—Mild	• Acute cholecystitis not meeting other severity criteria • Mild gallbladder inflammation, no organ dysfunction
Grade 2—Moderate	Acute cholecystitis with any of the following but no organ/system dysfunction: • Elevated white blood cell count (> 18,000/mL) • Palpable tender mass at right upper quadrant • Duration of complaints exceeding 72 h • Marked local inflammation (such as biliary peritonitis, pericholecystic abscess, hepatic abscess, gangrenous cholecystitis, emphysematous cholecystitis)
Grade 3—Severe	Acute cholecystitis with dysfunction of any one of the following organs/systems: • Cardiovascular dysfunction (hypotension requiring treatment with dopamine > 5 mg/kg/min of body weight or any dose of norepinephrine) • Neurological dysfunction (decreased levels of consciousness) • Respiratory dysfunction (ratio of PaO2/FiO2 < 300) • Renal dysfunction (oliguria, creatine > 2.0 mg/dL) • Hepatic dysfunction (PT-INR > 1.5)

Cited from Yokoe et al. [3].

For antimicrobial therapy, we preferred a combination of third-generation cephalosporins and metronidazole for grade 1 TG18 AC cases, while Tazobactam was administered as a combination therapy with piperacillin, with clear rationale for its choice in TG 18 Grade II and III patients. All patients were closely monitored, with early warning signs managed through a notification system for alarming signs. We also involved the medical team to address any correctable comorbidities. Bleeding tendency and coagulopathy were assessed and managed to ensure preparedness for invasive procedures, such as PC.

The primary aim of the study is the indication of percutaneous cholecystostomy in our institution. Secondary objective; 1.the success rate of PC; 2. morbidity rates after PC; 3.the proportion of patients treated with PC who undergo subsequent cholecystectomy; 4. Clinical Outcome and long term follow up of percutaneous cholecystostomy.

### PC technique

All procedures were performed using an ultrasound-guided transhepatic technique, with a 7 French or larger PC DUAN catheter inserted using an Amplatz guidewire, depending on the radiologist’s preference and the nature of the GB contents. At our institution, interventional radiologists prefer the transhepatic approach for several reasons: it reduces the risk of intraperitoneal bile leakage, minimizes catheter migration, and lowers the likelihood of intestinal injury. Although the transhepatic approach carries a higher risk of bleeding, this risk was low in our study due to the expertise of the interventional radiologists, who consistently performed the procedure under optimal coagulation status (INR ≤ 1.5).

Bile aspiration was confirmed, and samples were collected for analysis before the catheter loop was positioned within the GB. Its position was verified with contrast injection. The catheter was then secured to the skin and connected to a drainage bag.

### Post PC insertion care

Patients should remain bedridden for 4 h after the procedure. Clinical observation, pain assessment, and vital sign monitoring are required every 15 min for the first 2 h, every 30 min for the following 2 h, and then every 6 h thereafter. Analgesics should be administered as needed. The puncture site must be kept clean, with no signs of hematoma. Nurses should immediately notify the attending physician if there is suspicion of active bleeding at the puncture site or around the PC tube. If active bleeding is suspected, an urgent CTA should be performed.

The PC tube should be inspected frequently for kinks, especially if the dressing is wet or leaking. Catheter output over 24 h and the color of the fluid should be recorded each time the drainage bag is emptied, and the bag should be kept below the insertion site to allow proper drainage by gravity. Any complications that arise must be documented and addressed. The catheter should be handled gently during emptying and removed when no longer needed.

In most cases, PC catheter removal is planned based on the patient’s clinical progress, typically starting after 2 weeks of insertion. A cholecystogram is performed to confirm the free flow of bile from the gallbladder to the common bile duct (CBD). Once this is confirmed, the tube is clamped for 1 to 2 days while assessing the patient’s clinical status and conducting laboratory tests. If all clinical and laboratory findings are reassuring, the tube is then removed.

### Statistical analysis

Descriptive statistics in the form of mean and standard deviation for interval variables and frequency with percentages for categorical variables were calculated. Chi-square tests were applied to see the association between PC and study variable. One-way ANOVAs was performed to see mean differences among PC and all interval variables. P value < 0.05 (two tailed) was considered at a statistically significant level. SPSS 28.0 statistical package was used for the analysis.

## Results

97 patients were included in the study who underwent PC tube insertion for AC where Th primary aim of this study is to examine the indications for PC in AC management within our institution. Secondary aims include assessing PC feasibility, procedural safety, clinical outcomes, and long-term follow-up. 60 patients (61.9%) were male, the mean age of the cohort was 67.2 ± 15.5. 36 patients (37.1%) were Qatari (Native) and the rest were expats. 68 patients had diabetes mellitus, 81 had hypertension. There was no ASA I, but ASA II, III and IV were 10.3%, 47.4% and 41.2% respectively. High risk patients with CCI ≥ 5 represent 47.4% of the study cohort with mean of 4.27 ± 2.49.15 patients (15.2%) had acalculous cholecystitis, and the rest had calculous cholecystitis. Regarding TG 18 severity grading, most of the study population were grade II, was found in 56.4% of patients followed by grade I (32.3%).

All patients had ultrasound on admission, but Computed Tomography (CT) scan ordered for 39 patients (40.2%) and Magnetic Resonance Imaging (MRI) for 18 patients (18.6%). CT scans or MRIs were requested to confirm the diagnosis when ultrasound findings were unclear, when the CBD could not be adequately assessed, to improve the diagnosis of gangrenous cholecystitis, or to rule out other pathologies. The decision to perform these imaging studies was based on the treating physician’s judgment, as both modalities are readily available and easy to perform. Intensive care units (ICU) admissions were offered for 48 patients (49.5%) with a Mean ICU stay was 23.54 ± 29.77 days, with an average hospital stay (LOS) of 32.5 ± 35.5 days. The mean follow-up duration was 573.72 ± 597.99 days. The 30 days readmission rate with recurrent AC was 33.1% (32 patients). The mortality rate in our cohort was 34% (33 patients) which was unrelated to PC procedure and occurs during the post PC follow up time due to patients related medical condition. mortality cases were older in age with mean age of 70.42 ± 14.07 versus 65.56 ± 16.07 for the survivors and Mean CCI was 5.97 ± 2.8 which was going with high-risk status of these patients.

The mean length of PC stay was 32.6 days. Reinsertion of PC were required in eight patients, seven of them require two-time reinsertion and the last patient were offered five times PC reinsertions due to repeated AC. Complications related to PC was noticed in 9 patients (9.3%). 22 patients underwent cholecystectomy during follow up period after PC, and all done by laparoscopic approach. The mean duration between from PC insertion till cholecystectomy operation was 124.05 (median [IQR] 42 [17,173])

We correlated the study variables in the relation to population composition in the country, post PC cholecystectomy surgery and the reason behind PC.

Regarding demographic composition in Qatar: We noticed a significant age difference between the native population (Qatari) (≈ 10% of population) and expatriates (non-Qataris), which may be explained by the fact that immigrants are younger in age and hence more likely to work. We also discovered that Qataris had a higher incidence of diabetes and hypertension, which can be linked to their older age and the prevalence of these diseases in Qatar [16]. Accordingly, they had a higher CCI score (*P* = 0.001). one of the relationships that can explain why everyone gets the same health care no matter who they are (publicly funded primary health care settings). As we can see, Computed tomography (CT) scan abdomen and MRI were delivered similarly to all groups, but immigrants received more Magnetic resonance cholangiopancreatography (MRCP) (*P* = 0.047) despite the expense of this scan, and the death rate was insignificantly different between the two groups. Table 3 shows the relationship of the remainder variables with insignificant relation.

**Table 3:** Correlation regarding demographic composition of the country population

Patients’ characteristics	Non-Qatari (61 patients)	Qatari (36 patients)	*P*-value
**Demography**			
Gender (Male)	43 (71.1)	17 (28.3%)	0.033
Age	62.10 ± 15.368	75.89 ± 11.506	0.001
BMI	28.1226 ± 6.76984	30.2086 ± 6.41	0.138
**Comorbidities and risk**			
DM	37 (60.6%)	31 (86.1%)	0.008
HTN	46 (75.4)	35 (97.2)	0.005
CAD	31 (50.8%)	23 (63.9%)	0.211
CKD	14 (23.0%)	12 (33.3%)	0.265
Asthma	6 (9.8%)	4 (11.1%)	0.842
CLD	5 (8.2%)	1 (2.8%)	0.284
AKI requiring dialysis	15 (24.6%)	10 (27.8%)	0.729
CCI	3.54 ± 2.37	5.62 ± 2.15	0.001
Malignancy	5 (8.2%)	3 (8.3%)	0.981
Septic shock	20 (32.8%)	9 (25.0%)	0.418
History of abdominal surgery	7 (53.8%)	6 (46.2%)	0.468
ASA score (≥ 3)	51 (85%)	35 (97.2%)	0.079
**Radiology**			
US	47 (77.0%)	32 (88.9%)	0.147
Need for CT scan	25 (41.0%)	14 (38.9%)	0.839
Need for MRI/MRCP	15 (24.6%)	3 (8.3%)	0.047
**Laboratory**			
WBCS	17.92 ± 10.71	17.02 ± 8.48	0.670
HGB	11.35 ± 2.38	10.88 ± 2.25	0.339
PLT	254.48 ± 136.79	271.58 ± 140.84	0.558
Neutrophil	14.35 ± 7.63	12.63 ± 7.59	0.284
Lymphocyte	2.15 ± 7.38	1.55 ± 1.12	0.631
INR	1.39 ± 0.59	1.48 ± 1.32	0.664
BUN	9.39 ± 8.3	9.96 ± 5.72	0.717
Cr	155.55 ± 208.35	164.33 ± 137.25	0.822
NA	135.21 ± 4.43	134.89 ± 6.03	0.780
K	4.76 ± 4.39	5.19 ± 6.38	0.691
BIL T	34.85 ± 45.97	31.82 ± 49.49	0.761
BIL D	52.23 ± 50.61	61.49 ± 64.33	0.673
ALB	26.85 ± 8.21	26.28 ± 6.76	0.728
ALK	194.18 ± 192.5	187.28 ± 132.02	0.850
ALT	66.09 ± 102.07	94.8 ± 197.41	0.350
AST	86.08 ± 157.03	131.3 ± 278.76	0.319
CRP	198.13 ± 139.94	178.51 ± 120.94	0.493
Serum Lactate	2.7 ± 2.16	2.82 ± 1.97	0.809
Procalcitonin	14.38 ± 22.71	10.43 ± 21.84	0.509
**TG 18**			0.750
Grade 1	17 (34.0%)	9 (32.1%)	
Grade II	27 (54%)	17 (60.7%)	
Grade III	6 (12%)	2 (7.1%)	
**Hospital Course and Outcomes**			
ICU admission	31 (50.8%)	17 (47.2%)	0.732
ICU stay (days)	12.07 ± 15.03	18.56 ± 32.31	0.355
LOS (days)	26.4918 ± 27.33412	32.3056 ± 35.77934	0.370
Follow up (days)	469.32 ± 551.02	750.61 ± 639.66	0.024
30-day readmission	8 (13.6%)	6 (16.7%)	0.679
Number of PC catheter insertions	2 ± 0	3 ± 1.7	0.423
PC complications	7 (77.8%)	2 (22.2%)	0.332
Overall Mortalities	19 (57.6%)	14 (42.4)	0.437

In terms of PC indication, we analyzed this study cohort based on whether the indication is delayed presentation of AC or several comorbidities that carry a significant risk of operation. Considering the study variables, we discovered that; the patients with delayed presentation of AC are younger (mean age of 61.2 ± 13.17, *P* = 0.051) has a greater albumin level (*P* = 0.005), a higher hemoglobin level (*P* = 0.035), and a lower BUN level (*P* = 0.024). There were fewer comorbidities and significantly a smaller number of patients with a high ASA score (*P* = 0.001). In addition, as shown in Table 4, we demonstrated a lower need for ICU hospitalization (*P* = 0.002) and a lower number of deaths during the follow-up (*P* = 0.014). Mortality during follow-up was largely due to progressive comorbid conditions rather than procedural complications.

**Table 4:** Correlation of the study variables according to the indication of PC and post PC cholecystectomy

	Indication of PC			Post PC cholecystectomy		
	Delayed presentation (20)	multiple comorbidities (77)	*P*-value	No Surgery (73)	Cholecystectomy (22)	*P*- value
**Demography**						
AGE	61.2 ± 13.17	68.78 ± 15.77	*0.050*	68.96 ± 15.4	61.27 ± 14.74	*0.040*
Gender (Male)	11 (55.0%)	49 (64.5%)	0.436	45 (60.8%)	15 (68.2%)	0.531
BMI	27.11 ± 3.67	29.36 ± 7.21	0.057	29.24 ± 7.36	27.71 ± 3.38	0.174
**Vital Signs on Admission**						
TEMP	37.12 ± 0.51	36.94 ± 0.61	0.242	36.95 ± 0.59	37.1 ± 0.63	0.346
HR	90.8 ± 17.02	91.56 ± 17.66	0.864	91.44 ± 17.77	91.27 ± 16.69	0.969
RR	18.8 ± 1.7	20.56 ± 4.54	0.093	20.51 ± 4.54	19.14 ± 2.27	0.176
SBP	131.85 ± 21.83	127.22 ± 23.22	0.424	127.72 ± 22.11	129.73 ± 25.93	0.720
DBP	77 ± 12.2	69.51 ± 14.29	0.034	69.47 ± 13.56	76.45 ± 15.12	*0.041*
SAT	96.7 ± 3.67	97.22 ± 2.25	0.427	97.00 ± 2.77	97.50 ± 1.9	0.429
**Comorbidities and risk**						
DM	12 (60.0%)	56 (72.7%)	0.268	54 (72.0%)	14 (63.6%)	0.451
HTN	15 (75.0%)	66 (85.7%)	0.250	63 (84.0%)	18 (81.8%)	0.808
CAD	5 (25.0%)	49 (63.6%)	*0.002*	48 (64.0%)	6 (27.3%)	*0.002*
CKD	1 (5.0%)	25 (32.5%)	*0.013*	23 (30.7%)	3 (13.6%)	0.113
Asthma	0 (0.0%)	10 (13.0%)	0.089	8 (10.7%)	2 (9.1%)	0.831
CLD	0 (0.0%)	6 (7.8%)	0.197	6 (8.0%)	0 (0.0%)	0.171
AKI requiring dialysis	2 (10.0%)	23 (29.9%)	0.070	25 (33.3%)	0 (0.0%)	*0.002*
CCI	2.4 ± 1.6	4.75 ± 2.46	< 0.001	4.73 ± 2.49	2.68 ± 1.73	*< 0.001*
UTI	0 (0.0%)	11 (14.3%)	0.073	11 (14.7%)	0 (0.0%)	*0.049*
Malignancy	0 (0.0%)	8 (10.4%)	0.132	7 (9.3%)	1 (4.5%)	0.473
Septic shock	3 (15.0%)	26 (33.8%)	0.102	23 (30.7%)	6 (27.3%)	0.760
ASA Score (≥ 3)	12 (63.2%)	74 (96.1%)	*< 0.001*	72 (97.2%)	14 (18.9)	*< 0.001*
History of abdominal surgery	10 (15.0%)	3 (13.0%)	0.814	8 (10.7%)	5 (22.7%)	0.144
**Laboratory**						
WBCs	17.5 ± 7.28	17.6 ± 10.51	0.962	17.85 ± 10.71	16.67 ± 6.54	0.624
HGB	12.15 ± 2.15	10.92 ± 2.33	*0.035*	10.97 ± 2.41	11.87 ± 1.95	0.113
PLT	272.1 ± 128.16	257.9 ± 140.87	0.685	254.35 ± 138.03	282.91 ± 137.98	0.396
Neutrophil	14.28 ± 7.1	13.56 ± 7.79	0.708	13.66 ± 8.06	13.87 ± 6.04	0.911
Lymphocyte	1.48 ± 0.72	2.03 ± 6.59	0.708	2.1 ± 6.7	1.33 ± 0.72	0.593
INR	1.24 ± 0.23	1.47 ± 1.02	0.302	1.46 ± 1.0	1.32 ± 0.54	0.539
BUN	6.28 ± 4.26	10.46 ± 7.83	*0.024*	10.32 ± 7.39	7.13 ± 7.16	0.076
Cr	111.65 ± 141.17	171.1 ± 192.99	0.201	169.74 ± 195.02	121.55 ± 139.98	0.283
NA	134.15 ± 3.57	135.34 ± 5.36	0.352	134.9 ± 5.46	135.73 ± 3.33	0.506
K	4.02 ± 0.64	5.16 ± 5.79	0.384	5.19 ± 5.88	4.01 ± 0.58	0.352
BIL T	41.89 ± 69.56	31.6 ± 39.59	0.387	34.32 ± 51.4	31.7 ± 28.57	0.820
BIL D	67.12 ± 85.75	52.37 ± 45.77	0.566	58.38 ± 61.22	44.1 ± 9.74	0.579
ALB	30.87 ± 7.98	25.53 ± 7.24	*0.005*	24.99 ± 7.54	32.26 ± 5.1	*< 0.001*
ALK	145.21 ± 90.82	203.67 ± 185.8	0.176	200.02 ± 174.4	163 ± 163.35	0.377
ALT	52.21 ± 48.82	82.93 ± 159.76	0.411	66.05 ± 138.53	113.18 ± 163.08	0.182
AST	53 ± 78.12	115.35 ± 230.24	0.249	90.1 ± 194.05	145.52 ± 256.44	0.289
CRP	207.03 ± 128.82	186.56 ± 134.31	0.552	199.54 ± 140.59	160.6 ± 98.49	0.239
Serum Lactate	1.94 ± 0.94	2.95 ± 2.24	0.094	2.8 ± 2.26	2.56 ± 1.48	0.668
Procalcitonin	17.21 ± 23.24	12.26 ± 22.29	0.543	10.55 ± 18.04	24.24 ± 35.18	0.064
TG 18						
Grade 1	0 (0)	26 (42.6%)	0.001	23 (37.1%)	3 (18.8)	0.382
Grade II	16 (94.1%)	28 (45.9%)		33 (53.2%)	11 (68.8)	
Grade III	1 (5.9)	7 (11.5%)		6 (9.7%)	2 (12.5%)	
**Radiology**						
Need for CT scan	6 (30.0%)	33 (42.9%)	0.296	32 (42.7%)	7 (31.8%)	0.361
Need for MRI/MRCP	6 (30.0%)	12 (15.6%)	0.140	11 (14.7%)	7 (31.8%)	0.069
US finding						
GB stones	18 (90.0%)	61 (79.2%)	0.269	62 (82.7%)	17 (77.3%)	0.567
GB wall thickness	5.55 ± 1.47	5.59 ± 1.97	0.926	5.66 ± 1.92	5.32 ± 1.69	0.451
Pericholecystic fluid	41 (75.0%)	15 (53.2%)	0.079	41 (54.7%)	15 (68.2%)	0.259
CBD diameter	5.22 ± 3.44	5.64 ± 2.86	0.577	5.74 ± 3.18	4.91 ± 2.07	0.247
CBD stone	2 (10.0%)	5 (6.5%)	0.589	5 (6.7%)	2 (9.1%)	0.699
GB perforation	2 (10.0%)	6 (7.8%)	0.749	7 (9.3%)	1 (4.5%)	0.473
**Hospital Course and Outcomes**						
ICU admission	5 (25.0%)	43 (55.8%)	*0.014*	39 (52.0%)	9 (40.9%)	0.360
ICU stay (days)	8.40 ± 5.27	15.05 ± 23.64	0.538	15.78 ± 23.94	8.33 ± 14.26	0.378
LOS (days)	16.85 ± 12.33	31.71 ± 33.25	0.053	31.29 ± 32.15	19.64 ± 23.5	0.118
Follow up (days)	623.4 ± 505.21	560.82 ± 622.1	0.679	501.1 ± 611.7	821.32 ± 482.85	*0.026*
30-day readmission	2 (10.5%)	12 (15.8%)	0.563	10 (13.5%)	4 (19.0%)	0.528
Number of PC insertions	2 ± 0	2.43 ± 1.134	0.736	2.43 ± 1.134	2.00 ± 0	0.736
PC complications	1 (5.0%)	8 (10.4%)	0.459	8 (10.7%)	1 (4.5%)	0.384
Overall Mortalities	1 (5.0%)	32 (41.6%)	*0.002*	32 (42.7%)	1 (4.5%)	*< 0.001*

We compared patients who were offered surgery (cholecystectomy) to those who were not offered surgery (Table 4), and discovered that the surgery group was younger (*P* = 0.040), had a longer follow up period (*P* = 0.026), had a higher albumin level (*P* = 0.001), had less cardiac comorbidity (*P* = 0.001), had a lower ASA score (*P* = 0.001), had less acute kidney injury (AKI) at presentation (*P* = 0.002), lower CCI score (*P* = 0.001) and had fewer mortalities during follow up time (*P* = 0.001).

High risk patients represented the main bulk of the study cohort, about 86.7% of the patients, 16.3% of them were offered surgery during follow up time. when we compared high risk patients who operated to whom non operated, we found that operated cases were significantly had high albumin level(*P* = 0.004) (mean albumen was 30.98 ± 4.94 for operated patients versus 24.9 ± 7.4 non-operated cases), showing good kidney reserve as no one developed AKI during ED presentation of AC (*P* = 0.011) (zero cases for operated versus 24 patients who were non-operated (33.3%)).The most significant variable as the authors considered it the main cause of surgical indication was the readmission rate (64.3% of high risk cases in operated group versus 23.6% in non-operated one) (*P* = 0.002).

We found no relation between acalculous and calculous cholecystitis as main reason of AC with our variables apart from ultrasound finding of pericholecystic fluid was more in calculous cholecystitis and requirement for CT scan to confirm the diagnosis and exclude other pathology was more in acalculous type of AC as demonstrated in Table 5.

**Table 5:** Comparison between acute calculous and acalculous cholecystitis

	Acute Calculous Cholecystitis (73)	Acute Acalculous Cholecystitis (15)	*P*-value
**Demography and Clinical Status**			
Age	68.59 ± 15.05	61.17 ± 16.52	0.067
Male gender	48 (61.5%)	12 (66.7%)	0.685
BMI	28.87 ± 6.8	29 ± 6.32	0.937
ASA score (≥ 3)	80 (89.7%)	16 (88.8(%)	0.723
CCI	4.32 ± 2.48	4.06 ± 2.6	0.690
Septic shock	21 (26.6%)	8 (44.4%)	0.135
**Laboratory And Radiology**			
CRP	184.59 ± 126.06	216.37 ± 159.3	0.365
Serum Lactate	2.82 ± 2.21	2.37 ± 1.35	0.481
Procalcitonin	12.89 ± 22.77	13.32 ± 21.19	0.953
**US finding**			
GB wall thickness	5.57 ± 1.75	5.64 ± 2.36	0.889
Pericholecystic fluid	50 (63.3%)	6 (33.3%)	*0.020*
CBD diameter	5.43 ± 3.02	6.09 ± 2.79	0.399
CBD stone	5 (6.3%)	2 (11.1%)	0.479
GB perforation	8 (10.1%)	0 (0.0%)	0.159
Need for CT scan	28 (35.4%)	11 (61.1%)	*0.045*
Need for MRI/MRCP	14 (17.7%)	4 (22.2%)	0.658
TG 18			
Grade 1	25 (36.8%)	1 (10%)	0.191
Grade II	37 (54.4%)	7 (70%)	
Grade III	6 (8.8)	2 (20%)	
**Hospital Course and Outcomes**			
ICU admission	37 (46.8%)	11 (61.1%)	0.274
ICU stay (days)	13.33 ± 20.27	17.9 ± 30.03	0.575
LOS (days)	28.73 ± 32.13	28.28 ± 24.1	0.955
Follow up (days)	541.97 ± 542.6	713.06 ± 802.06	0.399
30 days readmission	11 (14.3%)	3 (16.7%)	0.798
Overall Mortalities	26 (32.9%)	7 (38.9%)	0.629

## Discussion

When cholecystectomy is contraindicated, PC is a suitable option for overcoming the acute phase of AC with a procedure that is minimally invasive. AC is one of the most common acute surgical emergency admissions, and the standard surgical technique was surgical treatment via laparoscopy. In circumstances where surgery is challenging, many surgeons abandon surgery to minimize bleeding and iatrogenic adjacent organ damage in delayed presentation of AC or in high-risk patients with substantial underlying comorbidities that carries higher anesthesia risk [17]. During COVID-19, one more indication was introduced because most surgical societies preferred treating AC conservatively through empirical antibiotic treatment with or without PC [18].

The mean age of the study was 67.2 ± 15.5, according to findings in the literature that with increasing age, gall bladder stones formation as the major cause of AC rise, and there is no consensus till date about treatment of the elderly population with a high risk of surgery that open the door for another alternative way of management [19].

Doğrul et al. compared patients with high and low risk based on comorbidities and found that patients with high risk had older age, more male gender, a high ASA score, higher mortality, a longer hospital stay, and a lower chance of cholecystectomy (*P* = 0.001). In this study, we showed that the high-risk patients with multiple comorbidities had an older age, a higher ASA score, a lower serum albumin level, and a higher BUN level. In addition, as shown in Table 4, they required more ICU hospitalization and a higher number of deaths during the follow-up [20].

Although acalculous cholecystitis was reported in this study (15.4% of patients), we found no statistical significance in most of the study data between acute calculous or acalculous cholecystitis as an etiology of AC. There is evidence in the literature that acalculous cholecystitis is associated with gall bladder wall ischemia or perforation, however we did not discover this in the present study. Another research found that 33% of patients got acute acalculous cholecystitis that required PC, which is in line with our findings in high-risk comorbid individuals (Table 5) [21].

The mean length of PC stay among those in the study was 32.6 days, based on clinical improvement of patient status and radiological proof of biliary system patency via cholocystogram. Past studies indicated removal after 4–6 weeks to avoid recurrence AC [21, 22], however other studies showed early removal within 7–12 days based on clinical and radiological evaluation [23].

PC is now widely available and has a high technical success rate; our IR team completed this PC with 100% success, which corresponds to the Society of Interventional Radiology statistics on technical success (97.9%) [23–25]. According to our data, all patients were relieved of AC sepsis-related symptoms following PC that was supported by the literature that AC symptoms improved in up to 90% of cases [8, 26]. In previous studies, the rate of interval cholecystectomy approached 60% [8, 22, 27, 28]. We demonstrated here a rate of interval cholecystectomy of 22.7% of patients with a mean follow up time of 574 days, which was considered low rate, and we related this to the fact that a high percentage (79.3%) of patients had multiple comorbidities, which carried a high risk of surgery. This is consistent with the findings of ER et al., who found a 13.6% rate of cholecystectomy and attributed it to the same cause [25].

Malik et al. found that patients who underwent cholecystectomy were younger with fewer comorbidities, less ICU requirements, less ICU stay, less LOS, lower CRP level on admission, and a higher conversion rate (70% of laparoscopy cases) when compared to patients who underwent PC alone. In our study, we discovered that the surgery group was younger (*P* = 0.040), had a higher albumin level on admission (*P* = 0.001), had a lower ASA score (*P* = 0.002), had less AKI at presentation (*P* = 0.002), and had fewer mortalities during follow up time and we don’t have any conversion as all cases managed by laparoscopy [29].

There is no definitive proof that PC can treat AC permanently, but its significance in improving the patient’s health state is clear. Others argue that PC should be considered the ultimate treatment of high-risk patients since the risk of recurring hospitalization with AC after PC is better than providing them surgery with a high mortality rate [22, 30]. In the literature, recurrent bouts of AC were reported in up to 25% of cases, but another research showed just a 3% of cases [25, 31]. Incidence of recurrence; in our study, around 30 patients (31%) experienced AC during the follow-up period. Several trials of decreasing AC recurrence after PC were stated that by percutaneous cystic duct stent insertion or fluoroscopy guided gallstone removal were other maneuvers that can be added to PC to decrease the recurrence of AC, especially in high-risk patients as a trial of making PC the definitive treatment in those people [32, 33].

In contrast, despite recurrent AC that was handled conservatively, most of this study population achieved long-term symptom management with PC alone, with just eight patients (8.2%) requiring reinsertion of PC without notable difficulties. Sanjay et al. reported that 13.2% of their study population required PC reinsertion, he additionally reported 22% readmission, a mean PC length of stay of roughly 43 days, a 13.2% PC complications rate, and 18 patients (33%) obtained cholecystectomy with the average follow-up period was 910 days [21].

We demonstrated complication rate of PC of 9.2% of this study cohort, Tuncer et al. found that PC problems occurred in 15.6% of the research sample, which is nearly double our result. In the literature, the complication rate ranged from 0 to 13%, indicating that the complications rate in this research were in reasonable range [34, 35].

In literature, PC mortality is as low as (0.36%), whereas mortality following PC cholecystectomy is at 0.96%. Cooper et al. reported a death rate of 43%, whereas another study found that 56% of their patients died during follow-up, with the same finding of a high ASA score in mortality cases [25, 36]. There was no mortality associated with PC or cholecystectomy in the study we conducted. Our overall mortality rate was 34% (33 patients), which we thought was a fair rate, especially since they had a high ASA and CCI score, which is close to the literature rate. The author believed that higher mortality rate due to higher CCI and other medical illnesses that explain longer hospital stays supported managing any additional AC in such a group of patients with PC, were more logical, as with any surgical intervention in such patients, we only put more stress on their already fragile bodies and decreased their survival chances. The limitations of this study were its retrospective design with inherent bias and limited data quality, as well as the small sample size; therefore, a prospective study with a larger population would be more helpful in confirming the study conclusions.

## In conclusion

PC is a successful alternative for AC in high-risk individuals and in cases with delayed presentation with a high surgical risk. When interval cholecystectomy is planned, PC shows excellent technical success and relieves the patient’s symptoms. We acknowledge that PC is a safe procedure, provided that radiological expertise is available. Future research should focus on optimizing criteria for patient selection, with a particular emphasis on integrating PC as part of a structured approach for high-risk AC management.

## Data Availability

Data will be made available upon request from the corresponding author.

## References

[1] Pisano M, Allievi N, Gurusamy K, Borzellino G, Cimbanassi S, Boerna D, Coccolini F, Tufo A, Di Martino M, Leung J, et al. 2020 World Society of Emergency Surgery updated guidelines for the diagnosis and treatment of acute calculus cholecystitis. World J Emerg Surg. 2020;15(1):61. 33153472 10.1186/s13017-020-00336-x PMC7643471

[2] Park MS, Yu JS, Kim YH, Kim MJ, Kim JH, Lee S, Cho N, Kim DG, Kim KW. Acute cholecystitis: comparison of MR Cholangiography and US. Radiology. 1998;209(3):781–5. 9844674 10.1148/radiology.209.3.9844674

[3] Yokoe M, Hata J, Takada T, Strasberg SM, Asbun HJ, Wakabayashi G, Kozaka K, Endo I, Deziel DJ, Miura F, et al. Tokyo guidelines 2018: diagnostic criteria and severity grading of acute cholecystitis (with videos). J Hepatobiliary Pancreat Sci. 2018;25(1):41–54. 29032636 10.1002/jhbp.515

[4] Wakabayashi G, Iwashita Y, Hibi T, Takada T, Strasberg SM, Asbun HJ, Endo I, Umezawa A, Asai K, Suzuki K, et al. Tokyo guidelines 2018: surgical management of acute cholecystitis: safe steps in laparoscopic cholecystectomy for acute cholecystitis (with videos). J Hepatobiliary Pancreat Sci. 2018;25(1):73–86. 29095575 10.1002/jhbp.517

[5] Friedman GD. Natural history of asymptomatic and symptomatic gallstones. Am J Surg. 1993;165(4):399–404. 8480871 10.1016/s0002-9610(05)80930-4

[6] Warttig S, Ward S, Rogers G, Guideline Development G. Diagnosis and management of gallstone disease: summary of NICE guidance. BMJ. 2014;349:g6241. 25360037 10.1136/bmj.g6241

[7] Hatzidakis AA, Prassopoulos P, Petinarakis I, Sanidas E, Chrysos E, Chalkiadakis G, Tsiftsis D, Gourtsoyiannis NC. Acute cholecystitis in high-risk patients: percutaneous cholecystostomy vs conservative treatment. Eur Radiol. 2002;12(7):1778–84. 12111069 10.1007/s00330-001-1247-4

[8] Alvino DML, Fong ZV, McCarthy CJ, Velmahos G, Lillemoe KD, Mueller PR, Fagenholz PJ. Long-term outcomes following percutaneous Cholecystostomy Tube Placement for treatment of Acute Calculous Cholecystitis. J Gastrointest Surg. 2017;21(5):761–9. 28224465 10.1007/s11605-017-3375-4

[9] Brunt LM, Deziel DJ, Telem DA, Strasberg SM, Aggarwal R, Asbun H, Bonjer J, McDonald M, Alseidi A, Ujiki M et al. Safe Cholecystectomy Multi-society Practice Guideline and State of the Art Consensus Conference on Prevention of Bile Duct Injury During Cholecystectomy. *Ann Surg* 2020, 272(1):3–23. 10.1097/SLA.0000000000003791 32404658

[10] Radder RW. Ultrasonically guided percutaneous catheter drainage for gallbladder empyema. Diagn Imaging. 1980;49(6):330–3. 7215096

[11] De Simone B, Chouillard E, Di Saverio S, Pagani L, Sartelli M, Biffl WL, Coccolini F, Pieri A, Khan M, Borzellino G, et al. Emergency surgery during the COVID-19 pandemic: what you need to know for practice. Ann R Coll Surg Engl. 2020;102(5):323–32. 32352836 10.1308/rcsann.2020.0097 PMC7374780

[12] Association of Surgeons of Great Britain and Ireland. Intercollegiate general surgery guidance on COVID-19 update. (2020). Accessed: December 21, 2020: https://www.rcsed.ac.uk/news-publicaffairs/news/2020/march/intercollegiate-general-surgery-guidance-on-covid-19-update

[13] Mayumi T, Okamoto K, Takada T, Strasberg SM, Solomkin JS, Schlossberg D, Pitt HA, Yoshida M, Gomi H, Miura F, et al. Tokyo guidelines 2018: management bundles for acute cholangitis and cholecystitis. J Hepatobiliary Pancreat Sci. 2018;25(1):96–100. 29090868 10.1002/jhbp.519

[14] ASA Physical Status Classification System. Last approved by the ASA House of Delegates on October 15, 2014. https://www.asahq.org/resources/clinical-information/asa-physical-status-classification-system

[15] Charlson ME, Pompei P, Ales KL, MacKenzie CR. A new method of classifying prognostic comorbidity in longitudinal studies: development and validation. J Chronic Dis. 1987;40(5):373–83. 3558716 10.1016/0021-9681(87)90171-8

[16] Syed MA, Alnuaimi AS, Zainel AJ, HA AQ. Prevalence of non-communicable diseases by age, gender and nationality in publicly funded primary care settings in Qatar. BMJ Nutr Prev Health. 2019;2(1):20–9. 33235953 10.1136/bmjnph-2018-000014 PMC7678476

[17] Antunes D, Lami M, Chukwudi A, Dey A, Patel M, Shabana A, Shams M, Slack Z, Bond-Smith G, Tebala G. COVID-19 infection risk by open and laparoscopic surgical smoke: a systematic review of the literature. Surgeon. 2021;19(6):e452–61. 33757651 10.1016/j.surge.2021.02.003 PMC7927587

[18] Dvorak P, Hoffmann P, Renc O, Dusek T, Rejchrt S, Slezak O, Vyroubal P. Percutaneous cholecystostomy in the management of acute cholecystitis – 10 years of experience. Wideochir Inne Tech Maloinwazyjne. 2019;14(4):516–25. 31908697 10.5114/wiitm.2019.84704 PMC6939213

[19] Melloul E, Denys A, Demartines N, Calmes JM, Schafer M. Percutaneous drainage versus emergency cholecystectomy for the treatment of acute cholecystitis in critically ill patients: does it matter? World J Surg. 2011;35(4):826–33. 21318431 10.1007/s00268-011-0985-y

[20] Dogrul AB, Oruc M, Ciftci T, Hayran KM, Abbasoglu O. Factors affecting interval cholecystectomy and mortality in percutaneous cholecystostomy patients. Ulus Travma Acil Cerrahi Derg. 2022;28(12):1696–700. 36453787 10.14744/tjtes.2022.84294 PMC10198308

[21] Sanjay P, Mittapalli D, Marioud A, White RD, Ram R, Alijani A. Clinical outcomes of a percutaneous cholecystostomy for acute cholecystitis: a multicentre analysis. HPB (Oxford). 2013;15(7):511–6. 23750493 10.1111/j.1477-2574.2012.00610.x PMC3692020

[22] Solaini L, Paro B, Marcianò P, Pennacchio GV, Farfaglia R. Can percutaneous cholecystostomy be a definitive treatment in the elderly? Surg Pract. 2016;20(4):144–8.

[23] Devane AM, Annam A, Brody L, Gunn AJ, Himes EA, Patel S, Tam AL, Dariushnia SR. Society of Interventional Radiology Quality Improvement Standards for Percutaneous Cholecystostomy and Percutaneous Transhepatic biliary interventions. J Vasc Interv Radiol. 2020;31(11):1849–56. 33011014 10.1016/j.jvir.2020.07.015

[24] Kirkegard J, Horn T, Christensen SD, Larsen LP, Knudsen AR, Mortensen FV. Percutaneous cholecystostomy is an effective definitive treatment option for acute acalculous cholecystitis. Scand J Surg. 2015;104(4):238–43. 25567854 10.1177/1457496914564107

[25] Er S, Berkem H, Ozden S, Birben B, Cetinkaya E, Tez M, Yuksel BC. Clinical course of percutaneous cholecystostomies: a cross-sectional study. World J Clin Cases. 2020;8(6):1033–41. 32258074 10.12998/wjcc.v8.i6.1033 PMC7103974

[26] Winbladh A, Gullstrand P, Svanvik J, Sandstrom P. Systematic review of cholecystostomy as a treatment option in acute cholecystitis. HPB (Oxford). 2009;11(3):183–93. 19590646 10.1111/j.1477-2574.2009.00052.x PMC2697889

[27] de Mestral C, Gomez D, Haas B, Zagorski B, Rotstein OD, Nathens AB. Cholecystostomy: a bridge to hospital discharge but not delayed cholecystectomy. J Trauma Acute Care Surg. 2013;74(1):175–9. discussion 179–180. 23271093 10.1097/TA.0b013e31827890e1

[28] Yeo CS, Tay VW, Low JK, Woon WW, Punamiya SJ, Shelat VG. Outcomes of percutaneous cholecystostomy and predictors of eventual cholecystectomy. J Hepatobiliary Pancreat Sci. 2016;23(1):65–73. 26580708 10.1002/jhbp.304

[29] Malik A, Seretis C. Use of percutaneous cholecystostomy for complicated acute lithiasic cholecystitis: solving or deferring the problem? Pol Przegl Chir. 2021;93(0):7–12. 35384856 10.5604/01.3001.0015.4211

[30] La Greca A, Di Grezia M, Magalini S, Di Giorgio A, Lodoli C, Di Flumeri G, Cozza V, Pepe G, Foco M, Bossola M, et al. Comparison of cholecystectomy and percutaneous cholecystostomy in acute cholecystitis: results of a retrospective study. Eur Rev Med Pharmacol Sci. 2017;21(20):4668–74. 29131247

[31] Jang WS, Lim JU, Joo KR, Cha JM, Shin HP, Joo SH. Outcome of conservative percutaneous cholecystostomy in high-risk patients with acute cholecystitis and risk factors leading to surgery. Surg Endosc. 2015;29(8):2359–64. 25487543 10.1007/s00464-014-3961-4

[32] Comin JM, Cade RJ, Little AF. Percutaneous cystic duct stent placement in the treatment of acute cholecystitis. J Med Imaging Radiat Oncol. 2010;54(5):457–61. 20976992 10.1111/j.1754-9485.2010.02207.x

[33] Kim YH, Kim YJ, Shin TB. Fluoroscopy-guided percutaneous gallstone removal using a 12-Fr sheath in high-risk surgical patients with acute cholecystitis. Korean J Radiol. 2011;12(2):210–5. 21430938 10.3348/kjr.2011.12.2.210 PMC3052612

[34] Kiviniemi H, Mäkelä JT, Autio R, Tikkakoski T, Leinonen S, Siniluoto T, Perälä J, Päivänsalo M, Merikanto J. Percutaneous cholecystostomy in acute cholecystitis in high-risk patients: an analysis of 69 patients. Int Surg. 1998;83(4):299–302. 10096746

[35] Tuncer K, Kilinc Tuncer G, Calik B. Factors affecting the recurrence of acute cholecystitis after treatment with percutaneous cholecystostomy. BMC Surg. 2023;23(1):143. 37231394 10.1186/s12893-023-02042-2 PMC10214586

[36] Cooper S, Donovan M, Grieve DA. Outcomes of percutaneous cholecystostomy and predictors of subsequent cholecystectomy. ANZ J Surg. 2018;88(7–8):E598–601. 29052940 10.1111/ans.14251

